# High‐quality draft genome sequence of *Gaiella occulta* isolated from a 150 meter deep mineral water borehole and comparison with the genome sequences of other deep‐branching lineages of the phylum *Actinobacteria*

**DOI:** 10.1002/mbo3.840

**Published:** 2019-04-11

**Authors:** Rita Severino, Hugo J. C. Froufe, Cristina Barroso, Luciana Albuquerque, Alexandre Lobo‐da‐Cunha, Milton S. da Costa, Conceição Egas

**Affiliations:** ^1^ Center for Neuroscience and Cell Biology University of Coimbra Coimbra Portugal; ^2^ Next Generation Sequencing Unit Biocant Cantanhede Portugal; ^3^ CIMAR/CIIMAR—Centro Interdisciplinar de Investigação Marinha e Ambiental Universidade do Porto Porto Portugal; ^4^ Laboratório de Biologia Celular Instituto de Ciências Biomédicas Abel Salazar, ICBAS, Universidade do Porto Porto Portugal

**Keywords:** *Actinobacteria*, deep mineral water aquifer, *Gaiellaocculta*, *Gaiellaceae*, genome

## Abstract

*Gaiella occulta *strain F2‐233^T^ (=CECT 7815 = LMG 26412), isolated from a 150 meter deep mineral water aquifer, was deemed a candidate for high‐quality draft genome sequencing because of the rare environment from which it was isolated. The draft genome sequence (QQZY00000000) of strain F2‐233^T^ is composed of approximately 3 Mb, predicted 3,119 protein‐coding genes of which 2,545 were assigned putative functions. Genome analysis was done by comparison with the other deep‐branching *Actinobacteria* neighbors *Rubrobacter radiotolerans*, *Solirubrobacter soli* and *Thermoleophilum album*. The genes for the tricarboxylic acid cycle, gluconeogenesis and pentose phosphate pathway, were identified in *G. occulta*, *R. radiotolerans*, *S. soli* and *T. album* genomes. Genes of the Embden–Meyerhof–Parnas pathway and nitrate reduction were identified in *G. occulta*, *R. radiotolerans* and *S. soli*, but not in the *T. album* genome. Alkane degradation is precluded by genome analysis in *G. occulta*. Genes involved in *myo*‐inositol metabolism were found in both *S. soli* and *G. occulta* genomes. A Calvin–Benson–Bassham (CBB) cycle with a type I RuBisCO was identified in *G. occulta* genome, as well. However, experimental growth under several conditions was negative and CO_2_ fixation could not be proven in *G. occulta*.

## 1 INTRODUCTION

Bottled mineral waters originate from aquifers that are generally tapped from boreholes and piped to plants for blotting. According to European Union law mineral water cannot be disinfected to remove or decrease the number of microorganisms (Anonymous, 2009). However, the water can be treated to remove unstable elements such as iron, manganese, sulfur, and arsenic or to (re)introduce carbon dioxide. Therefore, bottled mineral water contains a complex microbiota that, presumably, originates from the source. Quality control procedures are mandatory to monitor the presence/absence of indicator bacteria for fecal contamination, as well as surface water infiltration and pathogenic bacteria such as *Pseudomonas aeruginosa* (Anonymous, 2009). Heterotrophic plate counts are also monitored to record alterations in the number of colony‐forming units (CFUs).

The majority of studies on the microbial diversity of bottled water have been performed on still natural mineral waters using culture‐dependent approaches (Guillot & Leclerc, 1993; Morais & da Costa, 1990; Vachée, Mossel, & Leclerc, 1997). Microbial abundances estimated by CFUs indicate that heterotrophic bacteria number is low at source (around 10 CFU/ml) but increases to about 10^4^–10^5^ CFU/ml during storage at room temperature (Croville, Cantet, & Saby, 2011; Morais & da Costa, 1990; Warburton, 1993). More recently culture‐dependent and culture‐independent techniques were used together to determine the microbial diversity and abundances present at source of one mineral water, to assess microbial stability of the source over a 1 year period, and to examine the microbial dynamics after bottling throughout 6 months of storage of the mineral water in factory produced plastic bottles (França, Lopéz‐Lopéz, Rosselló‐Móra, & da Costa, 2015). In all cases, communities were largely dominated by *Bacteria* affiliated with the *Alphaproteobacteria*, *Betaproteobacteria, *and* Gammaproteobacteria.* Several isolates representing new species were also characterized and described from the aquifer and the bottled mineral water (Albuquerque et al., 2011; França, Albuquerque, & da Costa, 2015; Franca, Albuquerque, Sanchez, Farelaira, & da Costa, 2017; Leandro, França, Nobre, Rainey, & da Costa, 2013; Leandro et al., 2012). Among them, *Gaiella occulta* is the sole representative of the family *Gaiellaceae* of the order *Gaiellales* within the deep‐branching lineages of the phylum *Actinobacteria* and was deemed a candidate for high‐quality genome sequencing given the rare environment from which it was isolated. The phylum *Actinobacteria* comprises several deeply branching lineages that consist of species of the orders *Rubrobacterales*, *Solirubrobacterales*, *Thermoleophilales*, and *Gaiellales* (Foesel, Geppert, Rohde, & Overmann, 2016). The order *Gaiellales* and the family *Gaiellaceae* comprises only the species *G. occulta* strain F2‐233^T^ (Albuquerque et al., 2011).

### 1.1 Organism information

**Figure 1:**
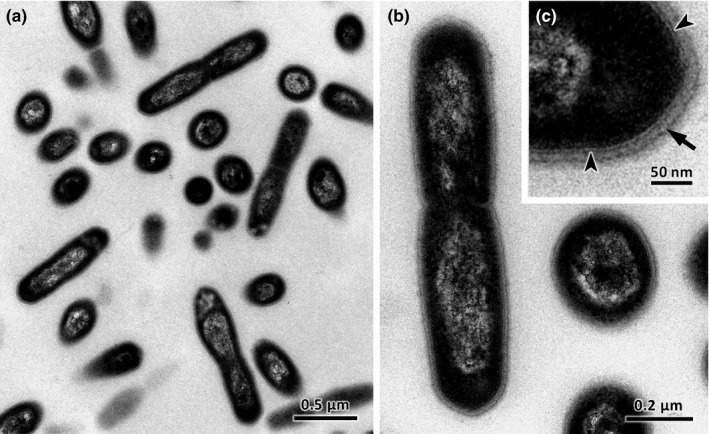
Transmission electron microscopy of *Gaiella occulta*. (a) Low amplification of a group of cells in longitudinal section and cross section. (b) Dividing cell with central septum. (c) Higher magnification showing a thin peptidoglycan layer (arrow) and outer leaflet of the cell membrane (arrow head)

*Gaiella occulta* is a nonpigmented, Gram‐negative staining, nonmotile, aerobic, and chemoorganotrophic, with an optimal growth temperature of 35–37°C, optimum pH for growth between 6.5 and 7.5, and was isolated from a 150 meter deep water aquifer in Portugal (Albuquerque et al., 2011; Foesel et al., 2016). The strain forms short rod‐shaped cells 1.0–3.0 µm in length by 0.3–0.5 µm in width (by transmission elctro microscopy) (Figure 1). Strain F2‐233^T^ can assimilate carbohydrates, organic acids, and amino acids. Nitrate is reduced to nitrite; long chain *n*‐alkanes are not used as carbon and energy source.

**Figure 2:**
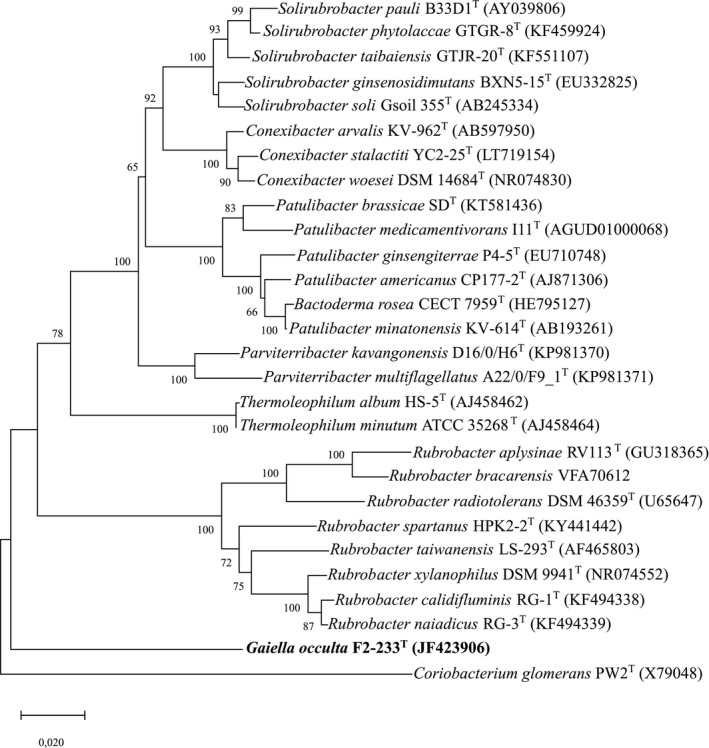
16S rRNA gene sequence phylogenetic tree. The position of *Gaiella occulta* within the radiation of the deep‐branching taxa of the *Actinobacteria* is shown. The scale bar represents two inferred substitutions per 100 nucleotides. The numbers at branching points of the neighbor‐joining tree represent bootstrap values from 1,000 replications

16S rRNA gene sequence comparisons show *G. occulta* to belong to the family *Gaiellacea* of the order *Gaiellales* which represents a deep branch lineage of the phylum *Actinobacteria.* The phylogenetic tree based on the 16S rRNA gene of strain F2‐233^T^ in the deep branching taxa of the *Actinobacteria* shows *G. occulta* F2‐233^T^ to form a distinct lineage separate from those of the orders *Rubrobacterales*, *Thermoleophilales,* and *Solirubrobacterales* (Figure 2).

## 2 MATERIALS AND METHODS

### 2.1 Growth conditions and genomic DNA preparation

Strain F2‐233^T^ was grown in 1 L Erlenmeyer flasks containing 300 ml of R2A medium (http://www.dsmz.de/microorganisms/medium/pdf/DSMZ_Medium830.pdf) at 37°C in a rotary water bath shaker until late exponential phase of growth for DNA extraction. To ascertain CO_2_ fixation and the enzymatic activity of RuBisCo, *G. occulta* was grown under aerobic and anaerobic conditions in sealed 50 ml serum ampules at 37°C containing 40 ml of a minimal medium previously described, supplemented with 0.02% yeast extract (Hahnke, Moosmann, Erb, & Strous, 2014). The medium contained (per liter) 0.5 g (NH_4_)_2_SO_4_, 0.5 g MgSO_4_.7H_2_O, 0.1 g CaCl_2_.2H_2_O, 6 g HEPES, 0.12 g K_2_HPO_4_, 0.04 g KH_2_PO_4_, and 1 ml trace element solution. Phosphate was added after sterilization. The trace element solution contained (per liter) 7.3 g Na_2_EDTA, 0.085 g CuCl_2_.2H_2_O, 0.72 g ZnSO_4_.7H_2_O, 2.5 g FeSO_4_.7H_2_O, 0.02 g MnCl_2_.4H_2_O, 0.242 g Na_2_MoO_4_.2H_2_O and 2 g NaHCO_3_. The headspace for anaerobic growth was flushed with N_2_ for 5 min. Formate, acetate and pyruvate were tested as sole carbon sources and mixtures of formate and acetate, or formate and pyruvate were used to improve growth. In order to test the utilization of hydrogen as electron acceptor, approximately 3 ml H_2_ was also added to anaerobic and aerobic ampules after sterilization and prior to inoculation. Cell density was measured at 610 nm.

Total genomic DNA was extracted following the method of Nielsen et  al. (Nielsen, Fritze, & Priest, 1995). Briefly, cells were lysed with a solution of lysozyme, guanidium thiocyanate, and sodium *n*‐lauryl sarcosine. DNA was extracted with chloroform:isoamyl alcohol (24:1, v:v), precipitated with isopropanol and washed with 70% ethanol, dried and resuspended in water. RNAse was included in the extraction process. DNA purity was assessed in a 1% agarose gel electrophoresis. DNA was quantified by fluorescence in the Invitrogen Qubit^®^ 2.0 fluorometer (Thermo Fisher Scientific, Carlsbad, CA).

### 2.2 Genome sequencing and assembly

Genomic DNA was prepared with the Nextera XT DNA Library Preparation Kit (Illumina) and sequenced using the paired‐end (PE) 2x300 bp V3 kit on the MiSeq^®^ (Illumina, San Diego) at the Next Generation Sequencing Unit, Center for Neuroscience and Cell Biology (CNC/Biocant, Cantanhede, Portugal). Sequenced reads were quality filtered with Trimmomatic (Bolger, Lohse, & Usadel, 2014) and assembled with SPAdes version 3.9.0 (Bankevich et al., 2012).

### 2.3 Genome annotation and analysis

The resulting contigs were annotated with PGP (Prokaryotic Genome Prediction) (Egas et al., 2014). Genome estimated completeness was verified with CheckM (version 1.0.7) based on lineage‐specific marker sets (Parks, Imelfort, Skennerton, Hugenholtz, & Tyson, 2015). Contamination was tested by CheckM for protein‐coding genes and RNAmmer version 1.2 (Lagesen et al., 2007) and Usearch61 (Edgar, 2010) (with the Greengenes database, version 13.8, ID threshold of 0.6 and an e‐value of 1e^−6^) for complete or partial 16S rRNA gene. The genome of strain F2‐233^T^ was compared with the available genomes of organisms of deeply branching orders of the *Actinobacteria*, namely *Rubrobacter radiotolerans* RSPS‐4 (NZ_CP007514.1, NZ_CP007515.1, NZ_CP007516.1 and NZ_CP007517.1), *Solirubrobacter soli* DSM 22325^T^ (NZ_AUIK00000000.1), and *Thermoleophilum album* strain ATCC 35263^T^ (NZ_FNWJ00000000.1), with GET_HOMOLOGUES using BLASTP and OrthoMCL (Contreras‐Moreira & Vinuesa, 2013). Orthologous genes were annotated against the Kyoto Encyclopedia of Genes and Genomes (KEGG) and assigned to metabolic pathways (sequence similarity cutoff e‐value of 1e^−5^) using KOBAS 2.0 (Xie et al., 2011).

One partial genome sequence of a *Gaiella* sp. (LSTI01000000) appearing in the database was amplified from a single cell recovered from soil. The sequence contains 995,360 bp with 1,036 genes coding for 915 proteins the vast majority of which are annotated as hypothetical proteins. The organism is closely related to *G. occulta* (95%) based on the phylogenetic analysis of a truncated 16S rRNA gene with 892 bp. The G + C ratio of the DNA is 66.4%.

## 3 RESULTS AND DISCUSSION

### 3.1 Genome properties

The F2‐233^T^ strain DNA sequence run generated 5,261,564 paired‐end reads of which 3,362,091 high‐quality reads remained after quality filtering. The average read length was of 197 bp. The *de novo* read assembly produced 34 contigs with an N50 size of 401,372 bp. The high‐quality draft assembled genome sequence consisted of 3,028,529 bp, with a sequencing depth of coverage of 520‐fold and a DNA G + C content of 71,65% (Table 1). The genome had a total of 3,167 genes, including 2,545 protein‐coding genes, 45 tRNA genes and three rRNA genes (a single copy of the genes 23S, 16S and 5S) (Table 1). CheckM estimated the genome to be near‐completion (97.84%) and the level of contamination to be extremely low (1.29%). No contamination was detected for 16S rRNA genes as tested by RNAmmer and Usearch61.

**Table 1:** Genome and annotation statistics for *Gaiella occulta* F2‐233^T^

Attribute	Value	% of Total
Genome size (bp)	3,028,529	100
DNA coding (bp)	2,785,809	91.99
DNA G + C (bp)	2,169,941	71.65
DNA scaffolds	34	
Total genes	3,167	100
Protein coding genes	3,119	98.48
RNA genes	48	1.51
Genes with function prediction	2,545	81.60
Genes assigned to COGs	1,718	54.24
Genes with Pfam domains	2,502	79.00
CRISPR repeats	1	

The draft genome sequence of *G. occulta* F2‐233^T^(=CECT 7815^T^ = LMG 26415^T^) has been deposited in the Short Read Archive (SRA) under the accession number SRR7537062 and the genome assembly under the accession number QQZY00000000.

### 3.2 Genome annotation

The draft genome comprised 2,545 genes with putative functions (~82% of total protein‐coding genes) and 1,718 genes allocated to the Clusters of orthologous grups (COG) functional categories (55% of total protein‐coding genes). The most abundant COG category was “Amino acid transport and metabolism” followed by “General function prediction only” and “Energy production and conversion” (Table 2).

**Table 2:** Number of genes associated with general COG functional categories

Code	Value	% of Total	Description
J	137	4.39	Translation, ribosomal structure and biogenesis
A	0	–	RNA processing and modification
K	150	4.81	Transcription
L	83	2.66	Replication, recombination and repair
B	1	0.03	Chromatin structure and dynamics
D	23	0.74	Cell cycle control, cell division, chromosome partitioning
V	19	0.61	Defense mechanisms
T	63	2.02	Signal transduction mechanisms
M	116	3.72	Cell wall/membrane biogenesis
N	36	1.15	Cell motility
U	43	1.38	Intracellular trafficking and secretion
O	65	2.08	Posttranslational modification, protein turnover, chaperones
C	170	5.45	Energy production and conversion
G	132	4.23	Carbohydrate transport and metabolism
E	263	8.43	Amino acid transport and metabolism
F	67	2.15	Nucleotide transport and metabolism
H	108	3.46	Coenzyme transport and metabolism
I	89	2.85	Lipid transport and metabolism
P	89	2.85	Inorganic ion transport and metabolism
Q	49	1.57	Secondary metabolites biosynthesis, transport and catabolism
R	240	7.69	General function prediction only
S	118	3.78	Function unknown
–	0	44.92	Not in COGs

### 3.3 Insights from the genome sequence

#### 3.3.1 Central metabolism

Genes coding for the enzymes of the Embden–Meyerhof–Parnas pathway (EMP), namely ATP‐dependent 6‐phosphofructokinase (EC 2.7.1.11, Gocc_2786, Gocc_2787) and fructose‐bisphosphate aldolase (EC 4.1.2.13, Gocc_1033) were identified in the genome of *G. occulta* as well as in the genomes of *R. radiotolerans* and *S. soli*. However, a fructose‐bisphosphate aldolase gene was not identified in the genome sequence of *T. album*, predicting that the organism cannot channel hexoses through this pathway.

The genes coding for 2‐keto‐3‐deoxygluconate‐6‐phosphate aldolase (EC 4.1.2.14) and 6‐phosphogluconate dehydratase (EC 4.2.1.12), characteristic of the Entner‐Doudoroff pathway were not found in the four genomes, indicating that glycolysis does not proceed through this pathway. The gene coding for the enzyme fructose‐1,6‐bisphosphatase (EC 3.1.3.11, Gocc_1034), required for gluconeogenesis, is present in the genome of *G. occulta* and in the genomes of *R. radiotolerans*, *S. soli,* and *T. album*. All putative genes coding for the enzymes of the pentose phosphate pathway and tricarboxylic acid cycle were identified in the genomes of *G. occulta*, *R. radiotolerans* and *S. soli* and *T. album*.

*Gaiella occulta* and *S. soli* utilize *myo*‐inositol as single carbon and energy source for growth (Albuquerque et al., 2011; Kim et al., 2007). Several genes coding for enzymes involved in the metabolism of *myo*‐inositol, namely *iolABCDEG,* were identified in *G. occulta* and *S. soli* genomes (Yoshida et al., 2008). However, the gene *iolJ* that codes for 6‐phospho‐5‐dehydro‐2‐deoxy‐D‐gluconate aldolase (EC 4.1.2.29) and leads to the formation of dihydroxyacetone‐phosphate and malonate semialdehyde was not identified in the genomes of these two organisms. This gene was also not found in the genomes of other bacterial strains capable of *myo*‐inositol catabolism such as *Clostridium perfringens*, *Legionella pneumophila,* and *Enterobacter aerogenes.* It is possible that the *iolJ* gene is replaced by a yet annotated aldolase gene (Berman & Magasanik, 1966; Kawsar, Ohtani, Okumura, Hayashi, & Shimizu, 2004; Manske, Schell, & Hilbi, 2016).

The genome predicts a Calvin–Benson–Bassham (CBB) cycle with a type I RuBisCO (EC 4.1.1.39, Gocc_0241 and Gocc_0242) but lacks sedoheptulose‐bisphosphatase (EC 3.1.3.37) that leads to the synthesis of sedoheptulose‐phosphate. This cycle is also predicted in the genome of *S. soli*. In *S. soli* DSM 22325^T^ the fructose‐1,6‐bisphosphatase/sedoheptulose‐1,7‐bisphosphatase gene is predicted to have a dual function, and this may also be the case for *G. occulta*. Other genes potentially involved in CO_2_ metabolism were identified in *G. occulta*, namely a formate dehydrogenase (EC 1.17.99.7, Gocc_1455), a putative hydrogenase (Gocc_1122 to Gocc_1127) and three carbon monoxide dehydrogenases (CODH, EC 1.2.5.3, Gocc_1071 to Gocc_1073, Gocc_1636 to Gocc_1638 and Gocc_2945 to Gocc_2947) (Shi et al., 2015). Several CODH genes were also identified in *R. radiotolerans* and *S. soli*. Growth was not observed under anaerobic conditions. Growth was observed under aerobic conditions in media containing acetate and pyruvate or combinations of formate plus acetate and formate plus pyruvate. The addition of hydrogen did not enhance growth under any condition examined. In all cases growth under aerobiosis was low and it was impossible to obtain enough cell mass to perform enzymatic assays for RuBisCO activity. We, therefore, predict but do not confirm CO_2_ fixation via the CBB cycle in this organism. A complete CBB cycle has also been identified in the genome of *Thermus* sp. NMX2.A1 although CO_2_ fixation through this pathway has not been confirmed in this organism (Müller et al., 2016).

A complete electron transport chain is predicted in *G. occulta* genome, namely NADH dehydrogenase (EC 1.6.5.3, Gocc_2267 to Gocc_2280), succinate dehydrogenase (EC 1.3.5.1, Gocc_1693 to Gocc_1695), cytochrome *bc1* (EC 1.10.2.‐, Gocc_3013 to Gocc_3015), and cytochrome *c* oxidase (EC 1.9.3.1, Gocc_0001, Gocc_0002, Gocc_0004). Homologs of *G. occulta* NADH dehydrogenase, succinate dehydrogenase, cytochrome *bc1* and cytochrome *c* oxidase genes were identified in the genomes of *S. soli* and *T. album*. Both *G. occulta*, *S. soli,* and *T. album* succinate dehydrogenase complex seems to lack the gene *sdhD*, which codes for the anchor subunit D, although the absence of this gene is not unusual (Horsefield, Iwata, & Byrne, 2004). In the genome of *R. radiotolerans*, we could not identify homologs for the following genes, NADH dehydrogenase *nuoE*, *nuoF* and *nuoG*, succinate dehydrogenase *sdhC*, and cytochrome *bc1* cytochrome‐*c* subunit. The ATPase of the type strains examined here are of the common bacterial F‐type.

*n*‐Alkanes with 13–20 carbons in length are utilized for growth of *T. album* (Zarilla & Perry, 1984). Genes involved in alkane degradation were identified in the *T. album* genome, namely an AlkG2‐type rubredoxin (WP_093117361.1), a putative flavin‐containing monooxygenase (EC 1.14.13.8, WP_093115507.1), and NAD(P)/FAD‐dependent oxidoreductase (WP_093116100.1), which shares 47.8% aminoacid sequence identity with *Acinetobacter* sp. *almA* (A5H9N6), involved in the degradation of long‐chain *n*‐alkanes (Rojo, 2009; Smits, Witholt, & van Beilen, 2003; Van Beilen et al., 2002). Homologs of *T. album* alkane‐degradation genes were also identified in *S. soli*. Genes for the metabolism of alkanes were not identified in the genome of *R. radiotolerans*, where alkane utilization was not examined, or in *G. occulta*, that does not utilize alkanes as growth sources (Albuquerque et al., 2011).

Genes coding for the uptake and reduction of nitrate were identified in the genomes of *G. occulta*, *R. radiotolerans,* and *S. soli*, namely the MFS‐type nitrate/nitrite transporter (*narK*/*nasA*, Gocc_2854) and the respiratory *nar*GHIJ nitrate reductase complex (EC 1.7.5.1, Gocc_2855 to Gocc_2859), although the reduction of nitrate to nitrite was not observed in *S. soli* (Kim et al., 2007). Genes coding for the two‐component system transduction pathway NarX/NarL identified in *G. occulta* (Gocc_1932 and Gocc_1933) were not identified in *S. soli* genome, but nitrate reductase expression does not seem to be altered by the absence of *narX* (Laub & Goulian, 2007; Sohaskey & Wayne, 2003). The *sox* genes, as well as other genes involved in sulfite oxidation/sulfate reduction, namely adenylylsulfate reductase (EC 1.8.99.2), dissimilatory sulfite reductase (EC 1.8.99.5) or sulfite dehydrogenase (cytochrome) (EC 1.8.2.1), were not identified in the *G. occulta* genome sequence or the other deeply branching *Actinobacteria* whose genome has been sequenced, precluding the utilization of reduced sulfur compounds as electron donors.

*Gaiella occulta* uses fructose, glucose, mannose, ribose, xylose, and *myo*‐inositol as single carbon source for growth (Albuquerque et al., 2011). The genes for ABC transport systems for ribose (Gocc_0079 to Gocc_0081; Gocc_2298 to Gocc_2300; Gocc_3068, Gocc_3069 and Gocc_3071), rhamnose (Gocc_0123 to Gocc_0125; Gocc_0231 to Gocc_0234), D‐xylose (Gocc_1293 to Gocc_1295), and raffinose/stachyose/melibiose (Gocc_1613 to Gocc_1616) were identified in *G. occulta* genome. Four unspecific sugar transport systems (Gocc_0123 to Gocc_0125, Gocc_0222 to Gocc_0224, Gocc_2321 to Gocc_2323 and Gocc_3042 to Gocc_3044), that may be involved in the transport of glucose, fructose, mannose, and *myo*‐inositol, were also identified. No PTS‐type transporters were identified in *G. occulta* genome. By in large, *S. soli* and *R. radiotolerans* have similar ABC transporters for sugars (Egas et al., 2014).

#### 3.3.2 Stress response

*Gaiella occulta* genome sequence has the key enzymes for the main DNA repair mechanisms, except for the mismatch repair pathway. Genes *mutS* and *mutL* were not encountered, as in many *Actinobacteria* and *Archaea* (Castañeda‐García et al., 2017). A gene coding for the endonuclease NucS (Gocc_1770) was identified, suggesting *G. occulta* may use the noncanonical mismatch repair pathway described recently for *Mycobacterium smegmatis* and *Streptomyces coelicolor* (Castañeda‐García et al., 2017). *Thermoleophilum album* and *S. soli* may also use this alternative mismatch repair pathway as they also lack homologs of *mutS* and *mutL* and have a *nucS* homolog.

*Rubrobacter xylanophilus* and *R. radiotolerans* accumulate the compatible solutes mannosylglycerate, trehalose, and low levels of di‐*myo*‐inositol‐phosphate generally involved in osmotic adapation in (hyper)thermophilic organisms that in the *Rubrobacter* spp. are constitutively accumulated (Empadinhas et al., 2007; Nobre, Alarico, Fernandes, Empadinhas, & da Costa, 2008). Genes coding for enzymes involved in the synthesis of mannosylglycerate or di‐*myo*‐inositol‐phosphate were not identified in *G. occulta*. In *R. radiotolerans*, trehalose synthesis can proceed via four pathways namely TpS/TpP, TreS, TreT, and TreY/TreZ (Egas et al., 2014; Nobre et al., 2008). In *G. occulta* only the genes coding for TpS/TpP and TreS were detected in the genome (*tpS*, EC 2.4.1.15, Gocc_2203, *tpP*, EC 3.1.3.12, Gocc_2154, *treS*, EC 5.4.99.16, Gocc_0097). The genes *tpS* and *tpP* were also identified in the genomes of *S. soli* and *T. album*.

Several genes involved in reactive oxygen species (ROS) detoxification in *R. radiotolerans* were identified in the genome of *G. occulta*, namely, two genes encoding peroxiredoxins (EC 1.11.1.15, Gocc_0321, Gocc_2238), which reduce hydrogen peroxide to water, genes coding for thioredoxin TrxA (Gocc_1414), thioredoxin reductase TrxB (EC 1.8.1.9, Gocc_2333), and glutaredoxin GrxC (Gocc_0876), involved in redox balance, three genes coding for LysR transcriptional regulators (Gocc_1704, Gocc_1792 and Gocc_0289) and one ABC‐type Mn^2+^/Zn^2+^ transport system (Gocc_1485, Gocc_1486) (Egas et al., 2014). In *T. album* genome we could not identify *lys*R transcriptional regulators or ABC‐type Mn^2+^/Zn^2+^ transport system genes.

## 4 CONCLUSIONS

The genome of *G. occulta*, the sole representative of the order *Gaiellales*, was sequenced, analyzed and compared to the existing genomes of the most closely related actinobacterial deep‐branching species *R. radiotolerans*, *S. soli,* and* T. album.* The main objective was to compare the results of the genome sequence analysis with the phenotypic characteristics of these organisms. Despite the phylogenetic distances between these strains obtained by 16S rRNA gene sequence analysis and different phenotypic characteristics, genome sequence analysis showed that many characteristics were shared among these organisms. With the exception of *T. album*, the genome analysis of the three other strains were similar with respect to the metabolism of hexoses and the central metabolism in general. We conclude that *T. album* cannot use sugars for lack of transporters and the lack of a fructose‐bisphosphate aldolase gene for metabolism of sugars. The genome of *G. occulta* and *S. soli* appear to predict CO_2_ fixation via the CBB cycle, however, CO_2_ fixation could not be proven. The type strain of *T.album* has genes that predict the hydrolysis of *n*‐alkanes and is known to be able to use only these substrates for growth. *S. soli* also possesses genes for the degradation of *n*‐alkanes, but growth on these substrates were not examined in this organism. *Gaiella occulta* does not possess the homologs found in *T. album* and does not grow on *n*‐alkanes. The results obtained in this study indicate that all organisms appear to be strict chemoorganotrophs and, for the most part, corroborate the phenotypes of these strains.

## CONFLICT OF INTERESTS

None declared.

## AUTHORS CONTRIBUTION

L. A. grew the organism, and extracted DNA. C. B. sequenced *G. occulta* genome. H. J. C. F. assembled, annotated and compared the genome sequence to other genome sequences available. A. L.‐d.‐C. performed Transmission Electron Microscopy. R. S. conducted anaerobic growth experiments and analyzed metabolic pathways. R. S., C. E. and M. S. d. C. wrote the manuscript.

## ETHICS STATEMENT

None required.

## Data Availability

The genome sequence of *G. occulta* F2‐233^T^ is publicly available in the SRA under the accession number SRR7537062 and the genome assembly under the accession number QQZY00000000.
